# ACCESS Open Minds at the University of Alberta: Transforming student mental health services in a large Canadian post‐secondary educational institution

**DOI:** 10.1111/eip.12819

**Published:** 2019-06-27

**Authors:** Helen Vallianatos, Kevin Friese, Jessica M. Perez, Jane Slessor, Rajneek Thind, Joshua Dunn, Jessica Chisholm‐Nelson, Ridha Joober, Patricia Boksa, Shalini Lal, Ashok Malla, Srividya N. Iyer, Jai L. Shah

**Affiliations:** ^1^ Office of the Dean of Students University of Alberta Edmonton Alberta Canada; ^2^ Department of Anthropology University of Alberta Edmonton Alberta Canada; ^3^ ACCESS Open Minds University of Alberta Edmonton Alberta Canada; ^4^ ACCESS Open Minds (Pan‐Canadian Youth Mental Health Services Research Network) Douglas Mental Health University Institute Montreal Quebec Canada; ^5^ Department of Psychiatry McGill University Montreal Quebec Canada; ^6^ Prevention and Early Intervention Program for Psychosis (PEPP) Douglas Mental Health University Institute Montreal Quebec Canada; ^7^ School of Rehabilitation, Faculty of Medicine Université de Montréal Montreal Quebec Canada; ^8^ Centre de recherche du Centre hospitalier de l'Université de Montréal (CRCHUM) Montreal Quebec Canada

**Keywords:** accessibility, intake, post‐secondary campus health services, service transformation, student mental health, youth mental health, Canada

## Abstract

**Aim:**

Demands for mental health services in post‐secondary institutions are increasing. This paper describes key features of a response to these needs: ACCESS Open Minds University of Alberta (ACCESS OM UA) is focused on improving mental health services for first‐year students, as youth transition to university and adulthood.

**Methods:**

The core transformation activities at ACCESS OM UA are described, including early case identification, rapid access, appropriate and timely connections to follow‐up care and engagement of students and families/carers. In addition, we depict local experiences of transforming existing services around these objectives.

**Results:**

The ACCESS OM UA Network has brought together staff with diverse backgrounds in order to address the unique needs of students. Together with the addition of ACCESS Clinicians these elements represent a systematic effort to support not just mental health, but the student as a whole. Key learnings include the importance of community mapping to developing networks and partnerships, and engaging stakeholders from design through to implementation for transformation to be sustainable.

**Conclusions:**

Service transformation grounded in principles of community‐based research allows for incorporation of local knowledge, expertise and opportunities. This approach requires ample time to consult, develop rapport between staff and stakeholders across diverse units and develop processes in keeping with local opportunities and constraints. Ongoing efforts will continue to monitor changing student needs and to evaluate and adapt the transformations outlined in this paper to reflect those needs.

## 1 INTRODUCTION

While a growing body of research speaks to the urgency and complexities of youth mental health, more work is needed to define and understand effective approaches for youth in Canada (Malla, Iyer, Shah, et al., 2018; Malla, Shah, Iyer, et al., 2018). ACCESS Open Minds (ACCESS OM) is committed to developing and evaluating the impact of a framework of care that can be implemented across diverse sociocultural contexts in Canada.

One of 14 such sites, the University of Alberta (UA) is the only setting within the ACCESS OM network centred in a post‐secondary educational institution. The mental health of post‐secondary students requires further investigation, as concern over a “crisis” in mental health for this segment of the population has escalated in popular discourse (CMAJ, 2017; Kadison & DiGeronimo, 2004; Lunau, 2012; Payne, 2017; Purdon, 2017). Studies have shown that the growth of mental health problems affecting post‐secondary students spans the globe (Eisenberg, Hunt, & Speer, 2013; Liu et al., 2017; Macaskill, 2013; Stallman, 2010; Storrie, Ahern, & Tuckett, 2010). The stressors of post‐secondary student life, especially for first‐year students, can exacerbate mental health difficulties, as students navigate financial constraints, academic and volunteer commitments, and new social networks—often for the first time. These concerns are mirrored in Canada, where post‐secondary students face similar stressors that affect their mental health alongside financial, academic, and physical wellness (Kirsh et al., 2016).

While the rate of help‐seeking among students is relatively low (Reavley, McCann, & Jorm, 2012b), more students are self‐disclosing mental illness and there is increasing use of campus mental health services both in Canada and internationally (Beiter et al., 2015; Gunnell, Kidger, & Elvidge, 2018; Hunt & Eisenberg, 2010; Lipson, Lattie, & Eisenberg, 2018; Mowbray et al., 2006). The post‐secondary context therefore provides a unique opportunity to both transform existing mental health services and foster adaptive and coping strategies that students will carry with them for life. The implementation of the ACCESS OM framework at UA is focused on first‐year students attending this large university, a group between the ages of 18‐25.

An important element for investigating the post‐secondary mental health landscape is to recognize the diversity between and within institutions. UA is a large research‐intensive university, with 18 faculties. Approximately 38 000 students attend UA, with most (81%) being undergraduates—although there are 500 graduate programmes offered as well. The student body is broad, with many students coming from rural and remote areas, including a growing number of First Nations, Métis and Inuit students, and a large contingent of international students (~21% of the total student population). In addition, there is an increasing diversity in ages of undergraduate students and a growing number of students who are themselves parents. Each of these student communities may have different levels of vulnerability to mental health problems and different coping and help‐seeking capacities (Currie, Wild, Schopflocher, Laing, & Veugelers, 2012; Jaworska, De Somma, Fonseka, Heck, & MacQueen, 2016; Popadiuk & Arthur, 2004; Reavley, McCann, & Jorm, 2012a). Together, this diverse and changing student body should impact upon how student services are designed, advertised and delivered (Reavley & Jorm, 2010).

### 1.1 Historical context

**Figure 1:**
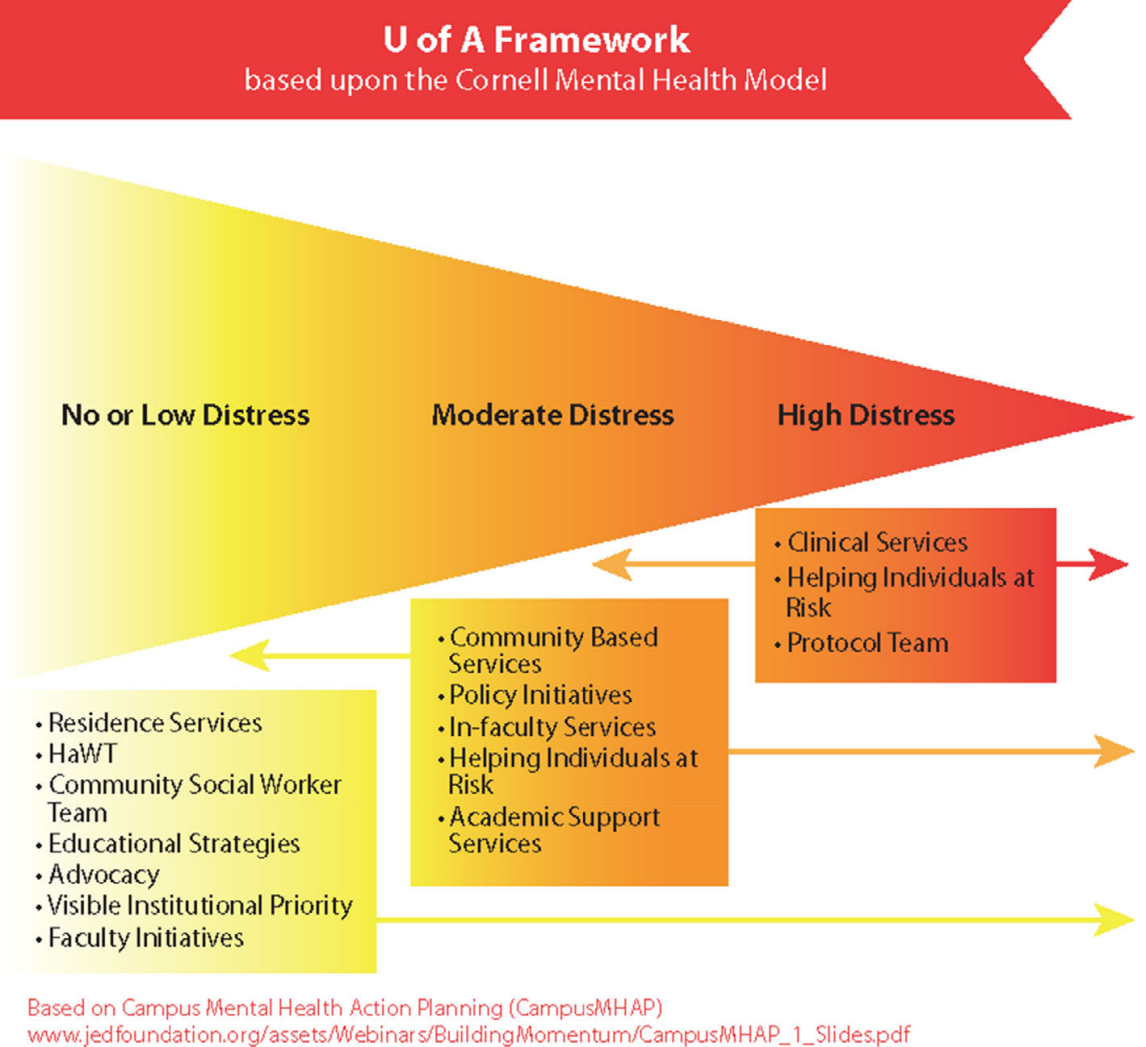
Visual of the University of Alberta reorganization prior to ACCESS Open Minds University of Alberta, based on the Cornell Mental Health Framework. *Source*: Everall, 2015 (p. 18)

In December 2012, the then‐Interim Dean of Students was commissioned by the university's Provost to lead a reorganization of campus mental health services to address rising demand (Figure 1). This reorganization was based on best practices in post‐secondary mental health services at that time (Cornell Mental Health Framework, n.d.; Eisenberg, Golberstein, & Hunt, 2009; Silverman, Underhile, & Keeling, 2008); a revised report was completed in 2015 (Everall, 2015). One outcome was the addition of community social workers (CSWs) as central figures in the service landscape. Rather than traditional one‐on‐one clinical social work, the work of CSWs aims to destigmatize mental illness, build mental health literacy and community capacity, educate students on coping and life skills, and ultimately to create opportunities that foster a culture of support and resiliency (along with destigmatizing access to formal services, if needed) while students learn to navigate the stressors that accompany university life.

A further outcome of the reorganization was a shift in the intake model at the university's Counselling and Clinical Services (CCS). Prior to the reorganization, intakes for counselling services functioned essentially on a first‐come, first‐serve basis. Given the increasing demand, a weeks‐long wait list quickly formed; those who did not have the capacity to call daily would not receive an appointment. In 2013, an intake model was implemented where students could book an intake consultation with a clinician (Mental Health Consultant, psychiatric nurse, etc.) within 72 hours of first contact, followed by referral to appropriate care or suggestions for other campus or community resources. This initial consultation essentially functioned as a triage point, allowing the team to identify and prioritize the most serious cases requiring immediate care. Typically, during peak stress times (e.g., final exams), staff were only able to see critical or urgent cases, leaving those with mild‐moderate concerns waiting.

While the mental health service landscape had significantly improved with the triage model in CCS and the community work of CSWs, demand continued to increase and students with mild‐moderate concerns were increasingly unable to access service. This was a lost opportunity for early intervention, as students typically could not access CCS services until their mental health problem had escalated. Furthermore, when students were referred to resources elsewhere (campus or community), there was no means of follow‐up. It is in the midst of these increasing demands on mental health services that ACCESS OM entered the scene, specifically in providing a framework to improve early intervention and enhance rapid access to appropriate care.

## 2 COMMUNITY MAPPING

**Figure 2:**
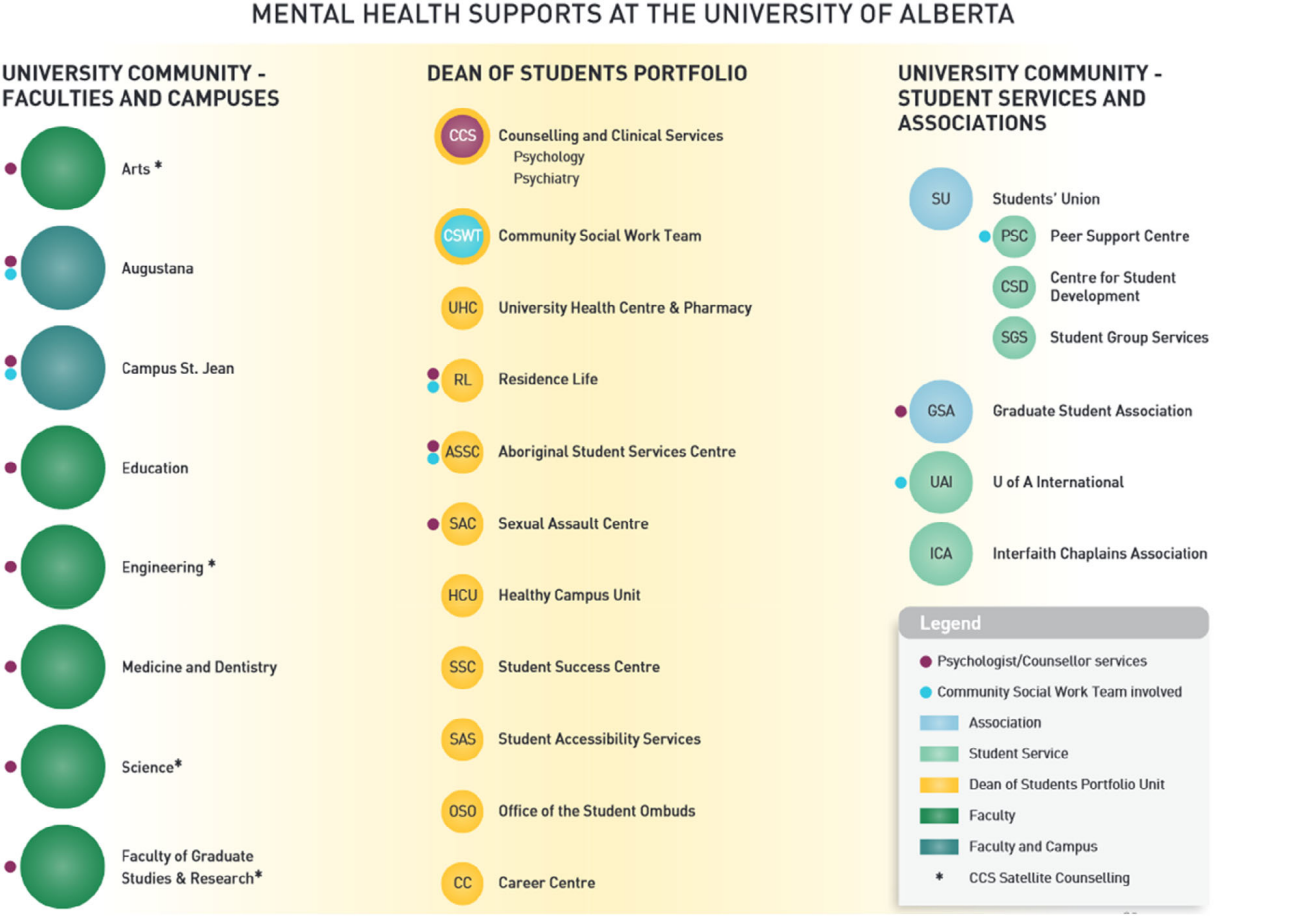
Illustration of campus and community resources that were previously identified. *Source*: Everall, 2015 (p. 23)

Figure 2 illustrates the range of on and off campus resources that were already known through environmental scanning activities prior to ACCESS OM (Everall, 2015). The main mental health service was CCS, consisting of psychologists and psychiatrists who specialized in psychotherapy and/or medication management, including specialists in attention deficit hyperactivity disorder, borderline personality disorder, and bipolar disorder. This service was located in the same space as most student services, but additional psychologists were embedded in faculties (Figure 2). The CCS team worked closely with, and received referrals from, the University Health Centre, Sexual Assault Centre and the CSW team, in addition to non‐health services and staff across campuses. Students could also self‐refer and schedule an initial consultation.

**Figure 3:**
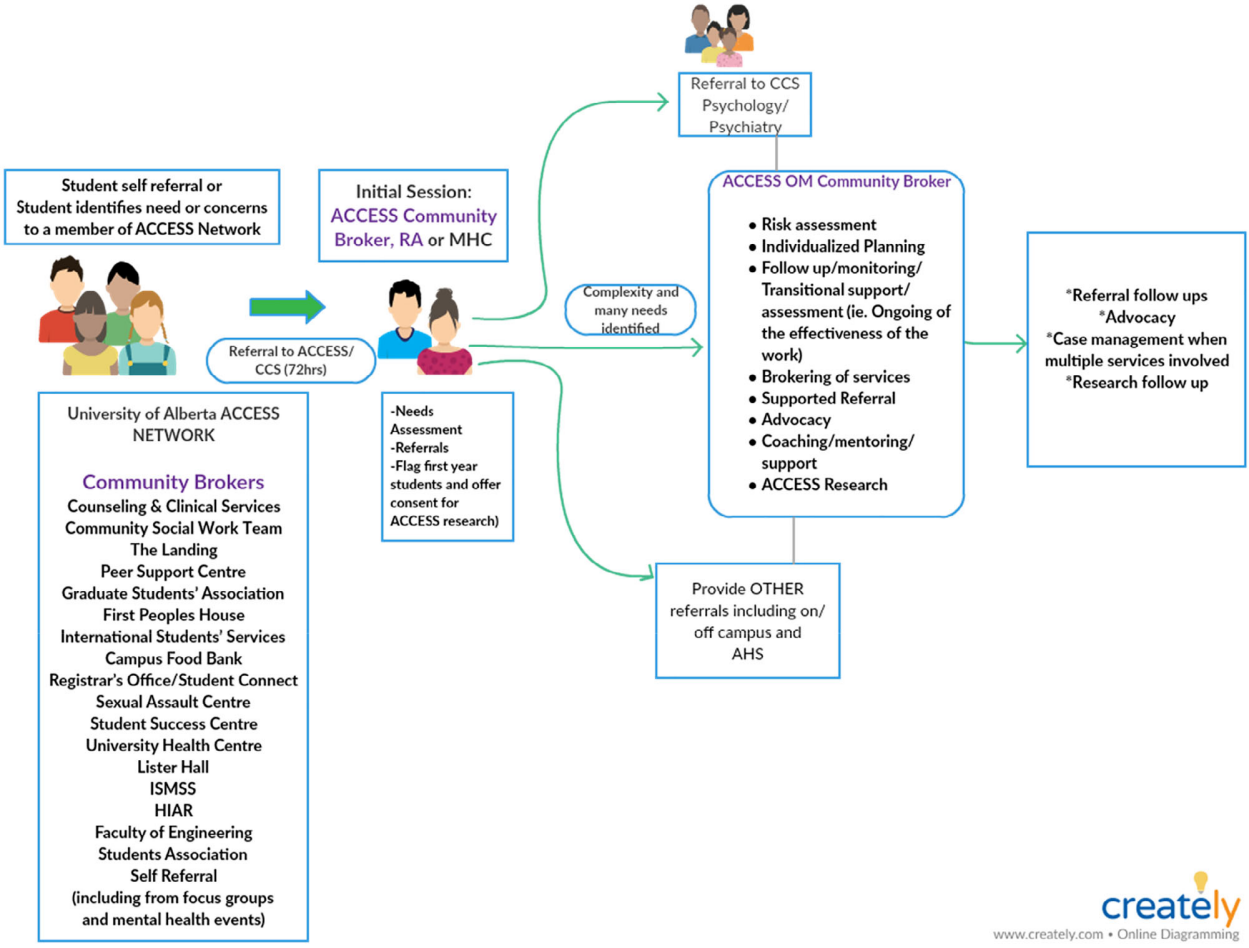
The ACCESS Open Minds University of Alberta (ACCESS OM UA) Network, highlighting the pathways and connections between all types of supports and services across campus, and the primary role of the ACCESS Clinicians in fostering these connections (called community brokers in this diagram)

As part of the implementation of the ACCESS OM project, from January to July 2017 a series of focus groups were undertaken across the university in order to assess students' beliefs and practices regarding their mental health, their current needs and their suggestions on how those identified needs were being or could be better met. Based on this, the first of the ACCESS Clinicians (both social workers) began outreach work with staff and other stakeholders within the university community in May 2017, ultimately forming the ACCESS OM UA Network. This network is led by the aforementioned Clinicians, and consists of delegates from various health, academic and non‐academic services distributed across campus, who in various ways encounter students experiencing mental health difficulties (see Figure 3 for a depiction of the range of services that comprise the Network). The network meets monthly to discuss referral processes, barriers to accessing all types of services and how to reduce these barriers, and trends in student concerns, and other issues. At a large institution, connecting diverse and disparate service providers across campus silos is a structural shift that would not have come about without ACCESS OM's tripartite focus on supporting students regardless of where they first seek help, then following this through to early identification and referral to mental health services when needed.

## 3 EARLY CASE IDENTIFICATION

Although students can self‐refer, the majority of students needing help are in fact, referred to the ACCESS Clinicians through the Network. A student in distress may be identified by any Network member; the aim is to ensure that no matter where a student turns for help, provision of appropriate services is facilitated. At an initial session, the student may meet with one of the two ACCESS Clinicians the ACCESS Evaluator, who is also a social worker, or CCS nursing staff. The initial session is held within 72 hours of referral, aiming to identify needs and to refer the student to appropriate services, which may include psychologists or psychiatrists working in CCS, other types of on‐campus supports (e.g., academic supports, campus food bank, etc.), and/or off‐campus resources (e.g., through Alberta Health Services [AHS]). The ACCESS Clinicians conduct any referral follow‐up and case management work.

Additional early case identification activities include: working with the ACCESS OM UA Network to improve referral processes and directly connect struggling or distressed students to ACCESS Clinicians consulting with non‐health service providers, faculty and staff; attending and/or supporting “student of concern” meetings that are held by the Dean of Students to ensure that appropriate referrals to mental health and other services are made; providing yoga classes to students and staff (e.g., Yoga for Mental Health classes); advertising to students through multiple modes of communication (e.g., emails, posters, slides on TVs located in public areas within UA buildings, attending events, etc.); and connecting with each FreshStart UA student (first‐year students who are struggling academically) to provide an initial assessment and then regular mental health check‐ins throughout the academic year.

## 4 RAPID ACCESS

Because of the ACCESS OM UA Network, students in distress can seek help not just at CCS but through multiple portals of entry on campus (Figure 3), where they will be directly referred to the ACCESS Clinicians. If the student accesses CCS first, an assessment of past or current mental health issues would be undertaken and available to the ACCESS Clinician. If the student was referred by a non‐health service Network partner, then the Clinicians themselves conduct a mental health assessment that includes screening for past or current conditions (and would be available to the CCS staff as needed). The ACCESS Clinicians strive to offer an initial session within 72 hours of referral, a goal that has been met except in situations where the student's schedule does not allow for a booking to occur within this timeframe (and the clinician is comfortable that the severity does not require immediate contact). Students may also refer themselves directly to ACCESS OM UA, via email, phone, or in‐person without a referral. The ACCESS Clinicians are mobile, have flexible hours, and meet students at a variety of locations based on students' preferences. All services are at no cost to the student.

ACCESS OM UA has established clear and flexible referral procedures with Network partners. With the goal of streamlining referrals, workflow, and reducing barriers to care, a review and coordination of intake processes and practices across different services took place in 2018 and continues to be evaluated. For example, in summer 2018, ACCESS Clinicians worked closely with CCS to create a shared intake process, ensuring appropriate information is available to all clinicians while reducing transitions between services.

## 5 APPROPRIATE CARE (IN 30 DAYS)

ACCESS OM sites aim to offer a direct entry point to services and to initiate follow‐up or specialized services within 30 days, if required. Based on the ACCESS OM assessment, the ACCESS Clinician will collaborate with the student to develop an appropriate care plan that may include connection to social supports, address basic necessities (food security, housing, etc.) and/or provide a warm hand‐off to clinical services in the case of crisis and imminent harm. The ACCESS Clinician will, if needed, directly connect a client to a psychologist or psychiatrist in CCS or other appropriate campus service(s) (e.g., sometimes via a priority appointment). They have also connected students to appropriate services in the community when specialist care is required (e.g., for eating disorders), including working with colleagues in the ACCESS OM Edmonton site.

Where the student is already connected to mental health services and was then connected to ACCESS OM UA, the problem is often that mental health services alone were not sufficient. In those cases, the ACCESS Clinician also offers assistance with connecting to non‐health services—also aiming for the 30‐day benchmark. Thus, other services such as academic supports, peer support, campus food bank, the international centre, Indigenous students' centre, Accessibility Resources and so on, are engaged. In other words, in order to support the mental health of students, we have learned that campuses need to offer clinical mental health services alongside addressing social factors impacting mental health.

## 6 CONTINUITY OF CARE BEYOND AGE 18

Since the vast majority of students are 18 or older when they begin their studies at UA, the ACCESS OM objective of eliminating age‐based transitions at this age does not apply to the UA context.

## 7 YOUTH AND FAMILY ENGAGEMENT

As part of ensuring that services are designed with students, a new ACCESS OM UA Youth Council was formed. Composed of UA students committed to supporting students' mental health and collaborating with the ACCESS OM UA team, they promote the ACCESS OM project and other wellness initiatives to the campus community. Since the beginning of the project, the Council has acted as a consulting body for the development of the site, initiatives that derive from the project or other UA wellness initiatives that require students' perspective. For instance, they provided feedback during the development of the protocol (e.g., testing length of time assessment tools took to complete, flow of online versions of the protocol, etc.). And in early 2018, one of the Youth Council co‐chairs initiated a conversation on a partnership with the Canadian Mental Health Association and the UA on improving the supports they offer to students through a 24/7 information and crisis phone service known as “211”; this project is currently underway.

ACCESS OM UA maintains a youth‐friendly physical space where the ACCESS Clinicians practice. The space is located across the hall from CCS, and in the same building as most other student services. The ACCESS OM UA team is located behind a glass storefront window, where students see an open space with couches, snacks, and ambient lighting and surrounding offices.

Youth and families/carers are considered equal partners in all aspects of the ACCESS OM project. The ACCESS OM UA service team is student‐centred in that clinicians work with students as partners in their own care, to identify a plan of care that is appropriate for the student. This may include family members, friends or other key people in the student's life, if desired. The notion of “carers” expands the concept of family to include supporters such as students' peers, friends or roommates.

## 8 WHAT IS SPECIAL ABOUT THE NATURE/PROCESS OF SERVICE TRANSFORMATION AT UA

Any transformation that involves a major shift in service delivery must identify barriers and facilitators that inhibit or promote that transformation. From a student's perspective, prior to ACCESS OM UA one would have first faced multiple, disconnected portals of entry when seeking help (CCS, CSW, academic advising, etc.) with potentially long wait lists. It was difficult for students to know which door to approach first; and because services were disconnected from one another, there was little continuity of care or support for students between services or (once accepted to a service) between appointments.

Since the initiation of ACCESS OM, students have a clear portal of entry to contact if they need help for mental health issues, and the opportunity to meet with someone to assess their needs within 72 hours of reaching out. Through the new Network, students are also able to access the mobile ACCESS OM UA team via multiple routes, or in situations where a rapid response (such as a student in crisis presenting at a service where no professionals can immediately see them) is needed. Referrals between services are also now managed as “warm hand‐offs” with the service providers managing and supporting transitions; the onus is no longer on the students to know where to go. Between appointments, students can always reach out to the ACCESS OM UA team for support; conversely, the team actively follows up with students in situations of missed appointments or potential disengagement.

From the perspective of service providers (who vary in their desire and capacity to shift practice), in order for substantive change to be embraced and sustained, staff must support and believe in the new model of delivery. The initial years of the ACCESS OM UA project were spent attempting to enhance relationships between diverse service providers using a participatory approach, in order to learn from their local experiences and to incorporate their ideas and address their concerns regarding implementation of ACCESS OM at UA. Arguably, it was when existing services began working together and saw the benefit of the ACCESS OM UA team in their daily work supporting individual students that this systems change took root.

Another contributor to success has been the type and characteristics of staff hired for the ACCESS OM UA team. The ACCESS Clinicians have played a critical role in not only providing exceptional services to students, but also in engaging and then working collaboratively with existing services to improve relationships and pathways to care. Given the very short timeframe of the project, retaining these Clinicians has ensured that the transformation has been led on the ground by a cohesive group, provided continuity in driving the on‐campus transformation and facilitated on‐going positive and trusting relationships between service providers.

## 9 RESEARCH AND EVALUATION

Routine evaluation is a critical part of the ACCESS OM project, in order to inform care for individual youth as well as to better understand the evolution and overall quality of service that is being provided. The hiring of the first ACCESS Clinician was instrumental in determining a streamlined protocol that integrated the ACCESS OM tools into provision of care that was not overly burdensome for the student or the service provider. Since then, a number of options have been identified to ease the burden of data gathering on clinicians, such as adding a dedicated Evaluator to the ACCESS OM UA team who could collect data in conjunction with follow‐up/check‐in calls with clients. Additional strategies involve the Evaluator conducting outreach activities in residences where first‐year students live, and/or other places where students congregate.

Data collection for local evaluation and national research purposes has been incorporated into the intake process. The ACCESS UA Evaluator has increased the capacity to conduct follow‐up interviews, critical to measuring effectiveness of supports and services over time. Qualitative data also continues to be gathered through participatory methods; feedback from diverse student voices is critical so that we can continue to best serve student needs into the future. There are limits to our evaluation activities; for example, while it is clear that there is a relationship between mental health and student academic success (Eisenberg et al., 2009), our institution or ACCESS OM UA does not conduct exit interviews to determine if mental health problems were a causal factor in a student's withdrawal or failure to complete a course of study. Discussions with campus administrators may result in future shifts on if, and how, such data could be gathered.

## 10 SUSTAINABILITY

The ACCESS OM UA service transformation utilizes a participatory, community‐based approach that is grounded in local knowledge. We have adopted a mixed methods data collection approach that incorporates metrics on local service use as well as qualitative data that captures multiple stakeholders' ideas, experiences and knowledge (e.g., through focus groups), in order to lay the foundations for a sustainable transformation. Such an approach requires time to allow for ideas to be tried and iterated, and for beliefs and practices to concurrently shift. Arguably, when service transformation follows this approach, stakeholders are more invested in the process and outcome of the transformation, and the transformation has meaning and value to all.

The service transformation shepherded by ACCESS OM includes multiple components that are now highly valued by university administration and integrated within the UA mental health service landscape, including the ACCESS Clinicians (particularly their model of care that incorporates follow‐up) and the Network. ACCESS OM UA leadership have been actively working with university, philanthropy and government decision‐makers to advocate for the sustainability of the ACCESS OM UA model.

## 11 COMMUNITY IMPACT

Since its inception, ACCESS OM UA has contributed to significant changes across the UA campus mental health service landscape. Over roughly one year, the ACCESS OM UA team has become the primary source for responding to, and supporting students in need. Referrals come from across the university, illustrating the importance of creating the ACCESS OM UA Network. For example, staff providing financial services now connect students directly to the ACCESS Clinicians when financial health conversations reveal potential underlying mental health issues.

It appears that the ACCESS Clinicians increasingly play a critical role in the community, as a safe, welcoming and mobile option to receive help on a variety of issues. For students who are struggling with mental health issues, this is often accompanied by a variety of other needs. Having a single service where one can receive assistance on all of these needs is valued by students.

## 12 CHALLENGES

The ACCESS OM UA Network and open referral system has reduced the barriers to accessing care on campus; however, as knowledge about the service and early identification of those in need increases (at the same time as stigma around mental health issues improves), the numbers of students dropping in for services has increased significantly. An iterative—and at times creative—process of problem solving and service transformation will be required to ensure that students can consistently access care within 72 hours. For example, ACCESS Clinicians may increasingly work with other student services to ensure that some needs are met in the short term (e.g., partnering with Peer Support Services or CSWs).

The local political‐economic context is also critical for understanding the process of change in service transformation. With post‐secondary institutions being guided towards a health promotion and illness prevention focus, long‐term specialist care is to be provided off‐campus, in surrounding communities. While this approach complements aspects of the ACCESS OM model, it requires ACCESS OM UA to continue to support students on‐campus—while also finding timely community resources that can support longer‐term needs for UA students. Informal supports (such as peer support) and bridging supports (such as interim counselling) are avenues through which to ensure that individuals do not fall through the cracks during such transitions, or the wait times that are sometimes associated with them.

Other challenges encountered include: (a) supporting sustainable and meaningful youth engagement, given students' many commitments including academic, volunteer and employment duties—the ACCESS OM UA team meets with the Youth Council leaders and the group as a whole to foster discussions on the importance of self‐care; (b) change management across multidisciplinary teams requires extensive consultations to ensure engagement and buy‐in; (c) finding innovative ways to educate and inform students' family members on issues and available supports in the post‐secondary context while also respecting confidentiality requirements—sharing information on students' health problems with parents is not possible unless consent has been provided by the student. One potential means of conversing with parents is to generically share what typical processes and supports are provided to students who are experiencing mental health problems; (d) managing expectations on the role of ACCESS OM UA, especially as demand for the service increases—this is particularly important in a context where funders' priorities can shift; and (e) navigating issues of confidentiality and disclosure—individual services or providers may not be able to share information within the ACCESS OM UA Network (Figure 3), which can contribute to delays in obtaining an assessment by the ACCESS Clinicians. As a result, the Network is working towards development of a shared form/process that would enable such disclosure across services (with the student's consent).

## 13 CONCLUSIONS

Research undertaken in diverse settings has reported on the range of stressors that combine to impact student mental health, and post‐secondary institutions across North America and elsewhere are grappling with how to address students' needs when resources are limited. In this paper, we have described the process and key features of mental health service transformation in a large post‐secondary institution—and in particular how the addition of the ACCESS Clinician and (through them) the Network of services has changed the mental health service landscape through a holistic and engagement‐focused model of care. Service transformation that is grounded in the principles of community‐based research is recommended as a means of incorporating local knowledge, expertise, and opportunities given the particular needs and features of a post‐secondary educational environment: the diverse nature of the student body, the breadth of locations across campus(es) where early identification is required, the coordination required with specialist mental health providers (e.g., CCS), and the transition to independence and decreased involvement of family members around this period. In sum, the ACCESS OM UA case study is an example of how to holistically approach mental health services, where the complicated intersections of a variety of issues—academic, social, financial, familial, etc.—that influence student mental health can be simultaneously supported in a more seamless manner. Future efforts will continue to evaluate the effectiveness of the transformations outlined in this paper and to monitor changing student needs.
